# Antioxidant Effect of Extracts from the Coffee Residue in Raw and Cooked Meat

**DOI:** 10.3390/antiox5030021

**Published:** 2016-07-04

**Authors:** Ji-Hee Kim, Dong Uk Ahn, Jong Bang Eun, Sun Hee Moon

**Affiliations:** 1 Department of Animal Science, Iowa State University, Ames, IA 50010, USA; jiheek@iastate.edu (J.K.); duahn@iastate.edu (D.U.A.); 2 Department of Food Science and Technology, Chonnam National University, Gwangju 500-757, Korea; jbeun@jnu.ac.kr

**Keywords:** extract of coffee residue (ECR), antioxidant, meat system

## Abstract

The residue of ground coffee obtained after the brewing process (spent coffee) still contains various functional components with high antioxidant capacity and health benefits, but no attempts have been made to use it as a resource to produce value-added food ingredients. This study evaluates the antioxidant activity of ethanol or hot water extracts from the residues of coffee after brewing. An extraction experiment was carried out using the conventional solid–liquid methods, including ethanol and water as the extraction media at different temperatures and liquid/solid ratios. The antioxidant activity of extracts was tested for total phenolic compound (TPC), 2,2-diphenyl-1-picrylhydrazyl (DPPH), and 2-thiobarbituric acid reactive substances (TBARS) using oil emulsion and raw/cooked meat systems. The DPPH radical scavenging activity of the ethanol extracts with heating (HEE) and without heating (CEE) were higher than that of the hot water extracts (WE). The highest DPPH value of HEE and CEE at 1000 ppm was 91.22% and 90.21%, respectively. In oil emulsion and raw/cooked systems, both the water and ethanol extracts had similar antioxidant effects to the positive control (BHA), but HEE and CEE extracts showed stronger antioxidant activities than WE extract. These results indicated that the ethanol extracts of coffee residue have a strong antioxidant activity and have the potential to be used as a natural antioxidant in meat.

## 1. Introduction

Lipid oxidation is recognized as a major cause of quality deterioration of meat products because it produces volatile compounds that can induce an off-flavor and it changes the color of meat [1]. Synthetic antioxidants such as the butylated hydroxyanisole (BHA), butylated hydroxytoluene (BHT), tert-bytylhydroquinone (TBHQ) and propyl gallate (PG) are commonly used in meat products to prevent oxidative changes [2]. However, synthetic antioxidants are reported to have carcinogenic effects [2], and, thus, consumers are concerned about the foods containing synthetic antioxidants. Now, the consumption of functional foods or antioxidant from natural sources has become a trend, and numerous attempts have been made to develop antioxidants from natural sources.

Natural antioxidants from plant origins are safe and can replace the synthetic ones. Herbs, bearberry, sunflower and many other plant extracts have been widely used in foods to improve their flavor and quality, and to extend their shelf-life [3,4,5,6,7]. Coffee is well known as a rich source of antioxidants that can reduce oxidative stress in humans. Recently, the consumption of coffee around the world has increased significantly due to its positive health effects. Thousands of tons of residues after brewing ground coffee at restaurants, cafeterias, and on a consumer level are produced annually in the U.S., but all of them are disposed of. However, significant amounts of antioxidants can remain in the residues. So, if they are properly recovered, there could be a high opportunity to use them as natural antioxidants [8]. In a recent study, the extracts from the residue of brewed coffee exhibited anti-inflammatory, anti-tumor and anti-allergic activities due to the presence of phenolic compounds such as chlorogenic acid, caffeine, caffeic acid, trigonelline and protocatechuic acid [6,9,10,11]. Chlorogenic acid, which is one of the most abundant phenolic compounds in the extract of coffee residue (ECR), and has been reported to have many beneficial functions, including hepatoprotective, hypoglycemic, anti-bacterial, antiviral, anti-inflammatory and anti-carcinogenic activities in humans.

Various extraction techniques have been applied to recover antioxidant compounds from natural and organic sources. Solvents such as methanol, ethanol, acetone, ethyl acetate, and their combinations have been used to extract phenolics from coffee or the coffee residues, often with different proportions of water [11,12,13,14,15,16,17,18]. Among these extraction methods, hot water and ethanol treatments were safe and the most commonly used extraction techniques. In other studies, roasted coffee residue was extracted with methanol, ethanol, and *n*-hexane, respectively, in a shaking incubator at 25 °C [6]. The results indicated that water extracts of roasted coffee residues showed the best antioxidant properties such as chlorogenic acid, caffeine and trigonelline, etc., which might be mainly attributed to the polyphenolic and nonpolyphenolic compounds in the extract [6]. Xu et al. [19] mention that subcritical water extraction (SWE) is an effective technology for the recovery of bioactive components from ground coffee. They reported that the amount of total phenolics extracted under the optimal extraction conditions was 88.34 mg gallic acid equivalent (GAE)/g SWE, and the antioxidant activity was 38.28 mmol Trolox equivalents (TEs)/100 g SWE. Zuorro et al. [12] reported that extraction of coffee with aqueous ethanol under mild temperature conditions also produced high amounts of phenolic compounds (21.56 mg GAE/g coffee). The combination of microwave and ethanol helped the extraction efficiency of phenolic compounds (399 mg GAE/g extract, dry matter) from coffee, and the extract (20 µg/mL) exhibited a high in vitro antioxidant activity [20]. Mussatto et al. [13] used various conditions, including methanol concentration, solvent/solid ratio, and extraction time to extract antioxidant compounds from spent coffee. They found the maximum amount of phonelic compounds (18 mg GAE/g) in the spent coffee extracts when 50% methanol was used at a ratio of 23 mL per g of spent coffee ground. Zhang et al. [21] demonstrated that the microwave-assisted extraction with 50% ethanol at 60 °C extraction showed higher chlorogenic acids value than other extraction methods, and the yield of chlorogenic acids rapidly reached to 6.14% within 5 min. Thus, phenol-rich extracts could be obtained from the ground coffee using an environmentally friendly and simple extraction procedure. Many other methods and conditions for efficient extraction of antioxidant compounds from coffee can be available, the extraction methods should be safe, cheap and efficient.

The objective of this study was (1) to investigate the antioxidant potentials of the extract from coffee residues; and (2) to evaluate the antioxidant effect of the extract from coffee residues in oil emulsion and raw/cooked meat systems.

## 2. Material and Methods

### 2.1. Extraction of Brewed Coffee Residues

The residues after brewing ground coffee were obtained from a local cafeteria and used as the raw material to extract antioxidant compounds. Extraction of coffee residues was performed using ethanol or water. For ethanol extraction, the coffee residue (70 g) was mixed with 700 mL of ethanol and heated (80 °C) or kept at room temperature for 1 h, and then filtered through a Whatman No. 1 filter paper. The residue was re-extracted using the same conditions and the filtrates were pooled. For hot water extraction (WE), the coffee residue (70 g) was extracted with 700 mL of distilled water in water bath at 80 °C and under the same conditions.

The water extract was frozen directly, but the ethanol extract was frozen after removing ethanol in the extract using a rotary evaporator (BUCH Rotavapor R-200, Postfach, Schweiz) under vacuum system. Both water and ethanol extracts were lyophilized in a freeze dryer (Labconco Corp., Kansas City, MO, USA) and stored until use.

### 2.2. Determination of Total Phenolic Compounds in the Extract

The total phenolic content in ECR was determined using the Folin-Ciocalteu’s reagent as described by Singleton et al. [22] with some modifications. A 200 µL ethanolic stock solution (1 mg/mL) was mixed with 4 mL of 10% sodium carbonate solution. After 5 min of reaction time, two hundred microliter of 50% Folin-Ciocalteau’s reagents was added to the mixture. After 30 min, the absorbance was measured at 750 nm. Tests were done in triplicate and presented as mg gallic acid equivalent per weight extraction sample (mg GAE/g ECR).

### 2.3. DPPH Radical Scavenging Activity

2,2-Diphenyil-1-picrylhydrazyl (DPPH) radical scavenging capacity was determined as described by Goffman et al. [23]. An ethanolic stock solution (0.4 mL) of each sample at different concentration was added to 1.6 mL of the DPPH solution (80 mg DPPH/L in 100% ethanol). After 30 min at room temperature, the absorbance was measured at 515 nm. Radical scavenging activity was calculated as follows:

% DPPH radical scavenging activity = (1 − sample absorbance/blank absorbance) × 100

### 2.4. Antioxidant Activity of ECR

Lipid oxidation was measured using the 2-thiobarbituric acid-reactive substances (TBARS) methods. The antioxidant effect of ECR in oil emulsion was determined using the modified method of Ahn et al. [24]. An oil-in-water emulsion containing 1 g of corn oil (HyVee Inc., Ames, IA, USA) and 100 µL of Tween 20 in 100 mL of Tris-maleate buffer (pH 6.8) was prepared by homogenizing them using a Brinkaman Polytron (Type PT 10/35; Brinkman Instrument Inc., Westbury, NY, USA) for 4 min in an ice bath at high speed. Samples for lipid oxidation assay was prepared by mixing 8 mL of the oil emulsion, 0.5 mL of 0.2% ascorbic acid and 0.5 mL of 200 ppm of Fe^+3^ (FeCl_3_), and 1 mL of ECR samples (500 or 1000 ppm) in a 50-mL test tube. After vortex-mixing, the mixture was incubated at 37 °C for 72 h. One mL of the mixture was withdrawn to determine 2-thiobarbituric acid-reactive substances (TBARS) value at different time durations of incubation. One milliliter of oil emulsion was mixed with 2 mL of thiobarbituric acid/trichloroacetic acid solution (20 mM TBA/15% TCA, *w*/*v*) in a disposable test tube (13 × 100 mm), and 50 µL of 10% butylated hydroxyanisole in 90% ethanol were added. After vortex-mixing the mixture was incubated in a 90 °C water bath for 15 min to develop pink color. Then, the samples were cooled for 10 min in cold water, vortex-mixed and centrifuged at 3000× *g* for 15 min at 5 °C. One mL of the supernatant was taken to measure the absorbance at 532 nm against a blank prepared with 1 ml ethanol and 2 mL TBA/TCA solution. The amounts of TBARS were expressed as mg of malondialdehyde (MDA) per L of emulsion.

The antioxidant effect of ECR on raw meat was determined following the method Ahn et al. [25]. Five grams of ground raw meat was added with 15 mL of distilled water, 50 µL of BHA and 1 mL of ECR extracts (1000 ppm) and homogenized by using a Brinkaman Polytron for 10–15 s at high speed. The homogenate was incubated in a 37 °C-water bath for 12 h. One milliliter of the raw-meat homogenate was taken out at 0, 1, 3, 6, and 12 h of incubation and TBARS was determined.

For the antioxidant effect of ECR on cooked meat, chicken thigh meats were trimmed off skin or visible fat and ground through a 3-mm plate twice, added with ECR, and then mixed for 1 min in a bowl mixer. Ground chicken patties (approximately 50 g) were prepared, vacuum packaged individually in oxygen-permeable bags (oxygen-permeable nylon/polyethylene bags; Koch, Kansas City, MO, USA), and then cooked in a 90 °C water bath (Isotemp®, Fisher Scientific Inc., Pittsburgh, PA, USA) until the internal temperature of the patties reached 75 °C. After draining the meat juice, the cooked patties were re-packaged in oxygen permeable bags and then stored in 4 °C cold room. Lipid oxidation of the cooked chicken patties was determined at 0, 1, 3 and 5 days of storage using the TBARS method as described above. The amounts of TBARS were expressed as milligrams of malondialdehyde (MDA) per kilogram of meat homogenate or meat.

### 2.5. Statistical Analysis

All results are presented as mean ± standard deviation (SD) and standard error of the means (SEM). Statistical analysis was performed using the SPSS for Windows (version 20, SPSS Inc., Chicago, IL, USA). All experiments were replicated three times (*n =* 3). Mean values were compared using the one-way analysis of variance (ANOVA) followed by Duncan’s multiple range test.

## 3. Results and Discussion 

### 3.1. Total Phenolic Compound Values of ECR

Phenolic compounds, one of the most widely occurring groups of phytochemicals, are of considerable physiological and morphological importance in plants and have strong antioxidant properties. In order to evaluate the potential antioxidant capacity of the extracts from the coffee residue, it was reasonable to determine the content of total phenolic in ethanol (HEE or CEE) and hot water extracts (WE).

The total phenolic compound values of ECR sample were 41.97, 35.51, and 28.10 mg/mL for HEE, CEE and WE, respectively. When the coffee residue was extracted using ethanol with heating (HEE), the amount of phenolic compounds was higher than the other two methods (ethanol extraction at room temperature and hot-water extraction) (Table 1). The amounts of phenolic compounds found in the ECR were as high as compared those of others [12,13,26]; some differences in phenolic compounds could be attributed to the coffee extraction process, roasting and variety of coffee products utilized. Mussatto et al. [13] reported that the use of methanol as organic solvent gave better extracting results than the use of only distilled water. This could be due to the lower solubility of phenolic compounds in polar water than the organic solvent [13]. Other researchers also reported that ethanol and methanol had better extracting capability for phenolic compounds from black mate tea, citrus peel extract and mashua than distilled water [13,27,28,29]. The high extraction capability of ethanol is related to the chemical structure of the phenolic compounds that contain hydroxyl, benzoic and ketone groups in their structure [30,31].

This result indicated that the residue of brewed coffee still contains a significant amount of phenolic compounds and that can be used as a source for the phenolic antioxidants.

### 3.2. DPPH Radical Scavenging Activity

Some methods are more effective and specific than others in evaluating the antioxidant activity of samples. Thus, it is strongly advisable to use more than one method to determine the antioxidant potential of a sample properly and to better interpret the results [32]. This study used DPPH and TBARS methods to determine the antioxidant activity of ECR in model systems as well as in meat.

The water extract (WE) showed a high DPPH radical scavenging activity at a high concentration (1000 ppm). At lower concentrations, however, the antioxidant activity was not significant different (*p* < 0.05). The ECR prepared with ethanol had higher DPPH radical scavenging activity than that with water. The DPPH radical scavenging activity of HEE and CEE were not significantly different (*p* < 0.05) at 250 to 1000 ppm levels (Table 2). The DPPH value of HEE and CEE ranged from 38.16 to 90.39% and 37.10 to 89.05% at a concentration 250 to 1000 ppm. However, WE of ECR showed relatively low radical scavenging activity of 12.03, 28.86 and 55.42% at 250, 500 and 1000 ppm level, respectively (Table 2).

These results indicated that all ECR have considerable DPPH radical scavenging capacity. The HEE and CEE both exhibited higher DPPH radical scavenging activity than the WE, with the ethanol extract with heating displayed the highest level of free radical scavenging activity. These results implied that ethanol and particularly heated ethanol extracted greater amounts of compounds with high antioxidant activity. Also, Krings et al.’s [33] results showed ethanolic extracts of some roasted are economic source of natural antioxidant in the by-product. Illy et al. [34] reported that coffee extracts were more active than cocoa or black tea extracts in delaying low-density lipoprotein oxidation.

### 3.3. Antioxidant Activity of ECR in the Oil Emulsion System

Table 3 illustrates the antioxidant activity of ECR on the TBARS values of oil emulsion system during 72 h at 37 °C. The stability of oil emulsion is one of the most important parts of this experiment. Surfactant mixtures, for example, tween-20 can be more effective at emulsification and stabilization of the oil emulsion. During the incubation time, there are no visible or physical change in oil emulsion samples. At 0 h, the TBARS values were found to be the same for all treatments and increased significantly with the increase of incubation time. The TBARS value of control (without extract sample) increased rapidly from 0.066 to 0.365 mg MDA/L of oil emulsion. However, all ECR exhibited significantly lower TBARS values than the control. The TBARS values of WE extract increased from 0.033 to 0.331 mg MDA/L (1000 ppm) during 72 h incubation time, but it was still significantly lower than the control (*p* < 0.05). The HEE and CEE showed similar antioxidant activity to BHA (50 ppm). Higher concentrations of HEE and CEE extracts, however, did not improve the antioxidant activity significantly between 500 and 1000 ppm. After 72 h incubation, HEE and CEE at 500 to 1000 ppm showed 75.6%–78.6% lower TBARS values than the control. HEE and CEE at 500 ppm was as effective as 50 ppm BHA, indicating that they can be good antioxidants for oil emulsion. These results demonstrated that HEE and CEE have a potent antioxidant activity in oil emulsion.

Franco et al. [35] reported that ethanol was better than any other solvents in extracting antioxidant compounds from plant materials. Our results also showed that water had lower extraction power than ethanol because most of the water-soluble antioxidant compounds were already extracted during brewing process. On the other hand, water extracts was exhibited higher antioxidant activities than the solvent extracts in previous studies [6,13]. Differences in raw material, including roasting conditions, bean composition, growing conditions, and brewing methods could have an effect on the amount of phonolics remaining in the coffee residues [6,15].

### 3.4. Antioxidant Activity of ECR in the Meat Systems

#### 3.4.1. Raw-Meat Homogenates TBARS Method

Table 4 shows the antioxidant activity of ECR in raw-meat homogenates. The TBARS value of control (without extracts) significantly (*p* < 0.05) increased during the first 3 h of incubation and then remained the same. The meat homogenate with BHA did not show any changes in TBARS during incubation. The TBARS of HEE and CEE increased significantly during the 1 h of incubation and then remained the same or decreased after 12 h of incubation. The TBARS of WE increased during the 3 h of incubation and then decreased after 12 h. The TBARS values of BHA were the lowest among the treatments, indicating that 50 ppm BHA had the stronger antioxidants than 500 and 1000 ppm of HEE, CEE or WE treatments. However, all other treatments also showed significant (*p* < 0.05) antioxidant effects during the incubation. For all the ECR (HEE, CEE and WE) 1000 ppm showed stronger antioxidant effect than 500 ppm. Wong et al. [36] demonstrated that the addition of vitamin E at 25–100 µg/g or herbal extract at 30 µg/g in cooked beef homogenate showed a concentration dependent inhibition of lipid peroxidation. These results suggested that the ECR can be effective in delaying lipid oxidation in raw meat.

Between the ethanol and water extracts, ethanol extracts showed stronger antioxidant effects at the same concentration because organic solvents are more efficient in extracting antioxidant compounds from the coffee residue. Between the high temperature (heating) or room temperature extracts, high temperature extract showed a stronger antioxidant effect than the room-temperature extract. Much literature has indicated that coffee residue still contains significant amounts of phenolic compounds [6,8,11,12,13]. Meat with >2.0 TBARS values can produce an off-flavor that common consumers can recognize [3]. The TBARS values of meat homogenate with ECR are <1.0 mg/kg, which are within the acceptable level.

#### 3.4.2. Cooked-Meat Model System Using TBARS Method

The antioxidant effect of ECR and the BHA in the cooked-meat (ground beef patties) are shown in Table 5. At the 0 day, the TBARS value of control was significantly higher than that of all other treatments. The TBARS of cooked meat rapidly increased during the storage, especially in the control. The cooked meat added with 140 ppm BHA nearly stopped lipid oxidation during the 5-day storage period. ECR treatments showed varying antioxidant effects depending upon the extraction methods used: HEE extract showed the strongest and WE extract showed the weakest antioxidant effects among the ECRs. All the ECR treatments maintained low-levels of TBARS values after 1 day of storage, but the TBARS values increased significantly after 3 days of storage. The antioxidant effect of 140 ppm BHA was significantly higher than any of the ECR values.

It is difficult to explain why ECR do not show such high antioxidant activity as that shown in oil emulsion or raw-meat homogenate systems. However, it seems that the oxidative power of cooked meat is much stronger than that of the antioxidant capacity of the ECR, and the oxidation could not be stopped with the ECR alone. Mc Carthy et al. [37] and Ahn et al. [38] reported that the extracts of natural sources showed strong antioxidant effects in vegetable oil, fat or protein model system [39]. Food ingredients such as tea catechins (0.25%), rosemary (0.10%) and sage (0.05%) were effective in reducing lipid oxidation in patties manufactured from previously frozen pork [37]. Lee et al. [40] showed that mustard leaf possesses antioxidant activity in foods because of its high content of phenolic compounds. Also, cloves and grape seed extracts had strong antioxidant effects in TBAR values in silver carp fillets [41]. The result of ECR in cooked meat systems was hardly different from other systems (oil emulsion or raw-meat homogenate). However, the ECR does not have strong enough antioxidant potential to prevent lipid oxidation of cooked meat; it still showed higher antioxidant activity than the control. These results indicate that these antioxidants activity delayed lipid oxidation in the cooked-meat patties during storage. 

## 5. Conclusions

This study indicated that ethanol and water extracts of coffee residue showed significant antioxidant activity and DPPH radical scavenging capacity. Among the three different extraction methods, HEE was the best method in extracting antioxidant compounds from the coffee residues. HEE was effective in preventing lipid oxidation in oil emulsion and raw meat systems, but was not strong enough to prevent oxidative changes in cooked-meat packaged in oxygen permeable bags for more than 3 days. This suggested that residues of coffee after brewing have the potential to be used as a source of natural antioxidants.

## Figures and Tables

**Table 1:** The total phenolic compound values of extract of coffee residue (ECR) samples.

Sample	Conc. (ppm)	TPC (mg GAE activity/g ECR)
HEE ^1^	1000	41.97 ^a^ ± 2.49
CEE		35.51 ^b^ ± 2.93
WE		28.10 ^c^ ± 0.76

^a–c^ Means with different letters in a column are significantly different between extraction methods (*p* < 0.05); ^1^ HEE: ethanol extraction with heating; CEE: ethanol extraction with room temperature; WE: hot water extraction.

**Table 2:** DPPH radical scavenging activity of ECR with different concentrations.

Sample	Conc. (ppm)			SEM ^2^
	250	500	1000	
HEE ^1^	38.16 ^ax^ ± 1.33	72.15 ^ay^ ± 1.37	90.39 ^az^ ± 0.14	7.66
CEE	37.10 ^ax^ ± 1.28	69.39 ^ay^ ± 0.67	89.05 ^az^ ± 0.74	7.58
WE	12.03 ^bx^ ± 2.76	28.86 ^bx^ ± 1.52	55.42 ^by^ ± 0.75	6.34
SEM	4.28	7.00	5.74	

^a,b^ Means with different lowercase letters in a column are significantly different between extraction methods (*p* < 0.05); ^x–z^: Means with different capital letters in a row are significantly different between sample concentrations (*p* < 0.05); ^1^ HEE: ethanol extraction with heating; CEE: ethanol extraction with room temperature; WE: hot water extraction; ^2^ SEM: standard error of mean.

**Table 3:** Antioxidant effect of extract of coffee residue on the 2-thiobarbituric acid reactive substances (TBARS) values (mg malondialdehyde (MDA)/L of oil emulsion) of oil emulsion model system.

Sample	Con. (ppm)	Incubation Time (h)						SEM ^2^
		0	6	24	30	48	72	
Control ^1^		0.066 ^av^	0.094 ^aw^	0.182 ^ax^	0.292 ^ay^	0.309 ^ay^	0.365 ^az^	0.027
BHA	50	0.031 ^bv^	0.034 ^cwv^	0.038 ^cw^	0.047 ^cx^	0.056 ^cy^	0.081 ^cz^	0.005
HEE	500	0.025 ^bv^	0.040 ^cw^	0.051 ^cx^	0.052 ^cx^	0.056 ^cy^	0.082 ^cz^	0.004
	1000	0.027 ^bv^	0.039 ^cw^	0.048 ^cy^	0.049 ^cy^	0.049 ^cy^	0.078 ^cz^	0.003
CEE	500	0.026 ^bx^	0.040 ^cx^	0.031 ^cx^	0.048 ^cy^	0.061 ^cy^	0.089 ^cz^	0.005
	1000	0.027 ^bv^	0.042 ^cw^	0.050 ^cx^	0.050 ^cx^	0.061 ^cy^	0.086 ^cz^	0.004
WE	500	0.034 ^bu^	0.075 ^bv^	0.145 ^abw^	0.222 ^bx^	0.284 ^by^	0.326 ^bz^	0.025
	1000	0.033 ^bv^	0.096 ^aw^	0.112 ^bw^	0.214 ^bx^	0.277 ^by^	0.331 ^bz^	0.026
SEM		0.003	0.005	0.012	0.020	0.024	0.026	

^a–c^ Means with different letters in a column are significantly different between extraction methods and concentration (*p* < 0.05); ^u–z^: Means with different letters in a row are significantly different between incubation times (*p* < 0.05); ^1^ Control: without extraction sample; BHA: 50 ppm BHA solution; HEE: ethanol extraction with heating; CEE: ethanol extraction with room temperature; WE: hot water extract; ^2^ SEM: standard error of mean.

**Table 4:** TBARS values (mg MDA/kg of raw-meat homogenates) of meat homogenates with different ECR samples during storage at 37 °C.

Sample	Conc. (ppm)	Incubation Time (hours)					SEM ^2^
		0	1	3	6	12	
Control ^1^		0.190 ^ax^	0.363 ^ay^	0.454 ^az^	0.436 ^az^	0.469 ^az^	0.027
BHA	50	0.150 ^b^	0.191 ^e^	0.193 ^e^	0.191 ^d^	0.192 ^e^	0.006
HEE	500	0.202 ^ay^	0.267 ^cz^	0.261 ^dz^	0.267 ^cz^	0.227 ^cdy^	0.007
	1000	0.202 ^ayx^	0.228 ^dz^	0.237 ^dz^	0.219 ^dzy^	0.196 ^dex^	0.004
CEE	500	0.202 ^ay^	0.313 ^bz^	0.304 ^cz^	0.351 ^bz^	0.298 ^bz^	0.014
	1000	0.202 ^ay^	0.272 ^cz^	0.256 ^dz^	0.258 ^cz^	0.215 ^dey^	0.008
WE	500	0.202 ^ax^	0.309 ^by^	0.377 ^bz^	0.363 ^bz^	0.284 ^by^	0.017
	1000	0.202 ^ax^	0.273 ^cz^	0.291 ^cz^	0.286 ^cz^	0.249 ^cy^	0.009
SEM		0.008	0.010	0.016	0.016	0.018	

^a–e^ Means with different letters in a column are significantly different between extraction methods and concentration (*p* < 0.05); ^x–z^ Means with different letters in a row are significantly different between incubation times (*p* < 0.05); ^1^ Control: without extraction sample; BHA: 50 ppm BHA solution; HEE: ethanol extraction with heating; CEE: ethanol extraction with room temperature; WE: hot water extraction; ^2^ SEM: standard error of mean.

**Table 5:** TBARS values (mg MDA/kg of cooked-meat patties) of cooked chicken patties with different ECR samples during storage at 4 °C.

Sample	Conc. (ppm)	Storage Time (Days)				SEM ^2^
		0	1	3	5	
Control ^1^		0.105 ^ax^	0.393 ^ay^	0.807 ^az^	0.790 ^az^	0.063
BHA	140	0.022 ^cy^	0.026 ^dy^	0.040 ^dz^	0.039 ^dz^	0.001
HEE	1000	0.025 ^cw^	0.076 ^cx^	0.197 ^cy^	0.263 ^cz^	0.020
CEE	1000	0.031 ^bx^	0.121 ^by^	0.380 ^bz^	0.396 ^bz^	0.034
WE	1000	0.029 ^bw^	0.128 ^bx^	0.396 ^by^	0.494 ^bz^	0.023
SEM		0.005	0.023	0.048	0.048	

^a–d^ Means with different letters in a column are significantly different between extraction methods (*p* < 0.05); ^w–z^ Means with different letters in a row are significantly different between incubation times (*p* < 0.05); ^1^ Control: without extraction sample; BHA: 140 ppm BHA solution; HEE: ethanol extraction with heating; CEE: ethanol extraction with room temperature, WE: hot water extraction; ^2^ SEM: standard error of mean.

## Abbreviations

The following abbreviations are used in this manuscript:

HEE: ethanol extraction with heating
CEE: ethanol extraction at room temperature
WE: hot water extraction
